# Prevalence of hyperuricemia in preeclampsia: A systematic review and meta-analysis of studies from low - and middle - income countries

**DOI:** 10.1371/journal.pone.0345152

**Published:** 2026-06-26

**Authors:** Fatima Abdirizak Muse, Theoneste Hakizimana, Sowda Abdikarim, Deqa Abdulsalam, Fardowso Dahir Warsame, Fatima Abdallah Noor, Luswata Herbert, Hamdi Jama, Akankwasa Prosper, Jackson Kakooza, Catherine Lewis, Fathi Ali Araye, Emmanuel Okurut

**Affiliations:** 1 Department of Obstetrics and Gynecology, Kampala International University Western Campus, Ishaka, Uganda; 2 Department of Surgery, Kampala International University Western Campus, Ishaka, Uganda; 3 Department of Surgery, St. Joseph’s Hospital Kitovu, Masaka, Uganda; 4 Department of psyciatry and mental health, faculty of clinical medicine and dentistry at Kampala International University Western Campus, Ishaka, Uganda; Universidad de Murcia, SPAIN

## Abstract

**Background:**

Hyperuricemia is a recognized biochemical finding in preeclampsia (PE), but the reported frequency varies across low- and middle-income countries (LMICs). A pooled estimate from LMIC settings may help clarify the extent of this finding in hospital-based populations.

**Objective:**

To estimate the pooled prevalence of hyperuricemia among women with preeclampsia in hospital-based studies from LMICs.

**Methods:**

A systematic review and meta-analysis was conducted according to PRISMA guidelines and registered in PROSPERO (CRD420251107624). PubMed, Scopus, Web of Science, and Lens.org were searched for observational studies published from 2010 to 2025 reporting hyperuricemia among women with preeclampsia in LMIC hospital settings. Prevalence estimates were pooled using a random-effects model with logit transformation. Heterogeneity was assessed using Cochran’s Q, Tau², and I². Sensitivity analyses included subgrouping by hyperuricemia threshold and leave-one-out analysis.

**Results:**

Eleven studies involving 1,099 women with preeclampsia from seven LMICs were included. The pooled prevalence of hyperuricemia was 53.47% (95% CI: 45.17% to 61.58%), with low-to-moderate heterogeneity (I² = 27.47%, p = 0.204). Included studies used different diagnostic thresholds for hyperuricemia, ranging from 5.0 to 7.0 mg/dL. All studies were hospital-based, and reporting of gestational age at the time of uric acid measurement was inconsistent.

**Conclusions:**

Hyperuricemia was common among women with preeclampsia in hospital-based LMIC studies. These findings describe prevalence only and should not be interpreted as evidence of diagnostic accuracy, prognostic performance, or clinical utility. Future studies should standardize diagnostic thresholds and report gestational timing of uric acid measurement more consistently.

## Introduction

Preeclampsia (PE) is a multisystem hypertensive disorder of pregnancy characterized by new-onset hypertension (blood pressure ≥140/90 mmHg) after 20 weeks of gestation, often with proteinuria or end-organ damage, such as impaired liver or kidney function, thrombocytopenia, or neurological symptoms [1,2]. Severe PE (blood pressure ≥160/110 mmHg) and eclampsia, characterized by new onset seizures or coma, are critical complications [1,3]. Globally, PE affects 2%–8% of pregnancies, contributing to 76,000 maternal and 500,000 fetal or newborn deaths annually [4]. In low- and middle-income countries (LMICs), the incidence is sevenfold higher (2.8% vs. 0.4% in developed countries), accounting for significant maternal mortality (e.g., 18% in Ghana, 30.7% in Rwanda) [2,5].

Hyperuricemia, defined as serum uric acid levels ≥375 µmol/L (≥6.3 mg/dL), is a hallmark of PE, often preceding hypertension and proteinuria [2,6]. Unlike normal pregnancies, where uric acid decreases due to enhanced renal clearance, preeclamptic pregnancies show elevated levels due to reduced tubular excretion and endothelial dysfunction [7]. Uric acid may contribute to PE pathogenesis by impairing placental remodeling, inducing oxidative stress, and promoting inflammation [4,8]. Despite its potential as a cost-effective biomarker, evidence on its prevalence and clinical significance in LMICs is inconsistent, with some studies linking it to adverse outcomes (e.g., preterm birth, low birth weight) and others questioning its predictive value [9]. Given the high burden of preeclampsia in LMICs and the fragmented nature of available evidence, this study aimed to estimate the pooled prevalence of hyperuricemia among women with preeclampsia in hospital-based LMIC studies.

Although hyperuricemia in preeclampsia has been described in individual studies, especially from hospital settings, a pooled estimate focused on LMIC populations remains limited. The contribution of this review is not to establish diagnostic or prognostic utility, but to quantify how commonly hyperuricemia is reported in women with preeclampsia across LMIC hospital-based studies and to identify methodological features, including threshold variability and incomplete reporting of gestational timing, that affect interpretation of this evidence base.

## Methods

### Protocol and registration

This systematic review and meta-analysis adhered to the Preferred Reporting Items for Systematic Reviews and Meta-Analyses (PRISMA) guidelines [10] and was registered with PROSPERO (CRD420251107624). Ethical approval was not required as only published data were analyzed.

### Database and search strategy

The PICO framework was used to develop the study question: Population (P): pregnant women with PE in LMIC hospitals; Intervention (I): not applicable; Comparison (C): women with or without hyperuricemia; Outcome (O): pooled prevalence of hyperuricemia in PE. A comprehensive literature search was performed using PubMed, SCOPUS, Web of Science, Lens.org, and Google Scholar of relevant studies from January 2010 to May 2025, using terms for hyperuricemia (e.g., “hyperuricemia,” “serum uric acid”), preeclampsia (e.g., “preeclampsia,” “gestational hypertension”), LMIC settings (e.g., “low-income,” “Sub-Saharan Africa”), and pregnancy (e.g., “pregnant women”). Search strings are provided in S1 File. No language restrictions were applied during the search. However, only studies published in English or available in English translation were included in the final review.

### Study selection

Eligible studies were observational, peer-reviewed, conducted in LMIC hospitals, and reported primary data on hyperuricemia prevalence in PE. Exclusions included randomized clinical trials, case reports, systematic and narrative reviews, non-LMIC or community-based studies, and non-English texts without translations. Two reviewers independently screened titles and abstracts using Rayyan, with full-text assessments by a third reviewer to resolve disputes.

### Data extraction and quality assessment

Data were extracted using a standardized Excel form, capturing study characteristics, sample size, hyperuricemia definition, number of hyperuricemia cases, and prevalence estimates. Study quality was assessed using the Joanna Briggs Institute (JBI) checklist, with scores ≥6 indicating high quality (S1 Table).

### Statistical analysis

Prevalence proportions were logit-transformed and pooled using a random-effects model with restricted maximum likelihood estimation in Jamovi v2.6.44 using the MAJOR module. The pooled estimate was back-transformed and presented as a percentage with 95% confidence intervals. Statistical heterogeneity was assessed using Cochran’s Q, Tau², and I² statistics. Prespecified subgroup analysis was performed according to the diagnostic threshold used to define hyperuricemia. Leave-one-out sensitivity analysis was also conducted to evaluate the influence of individual studies on the pooled prevalence estimate and heterogeneity. Because this review synthesized single-group prevalence estimates rather than comparative intervention effects, formal assessment of publication bias and small-study effects was interpreted cautiously and treated as exploratory.

## Results

### Study selection

**Fig 1:**
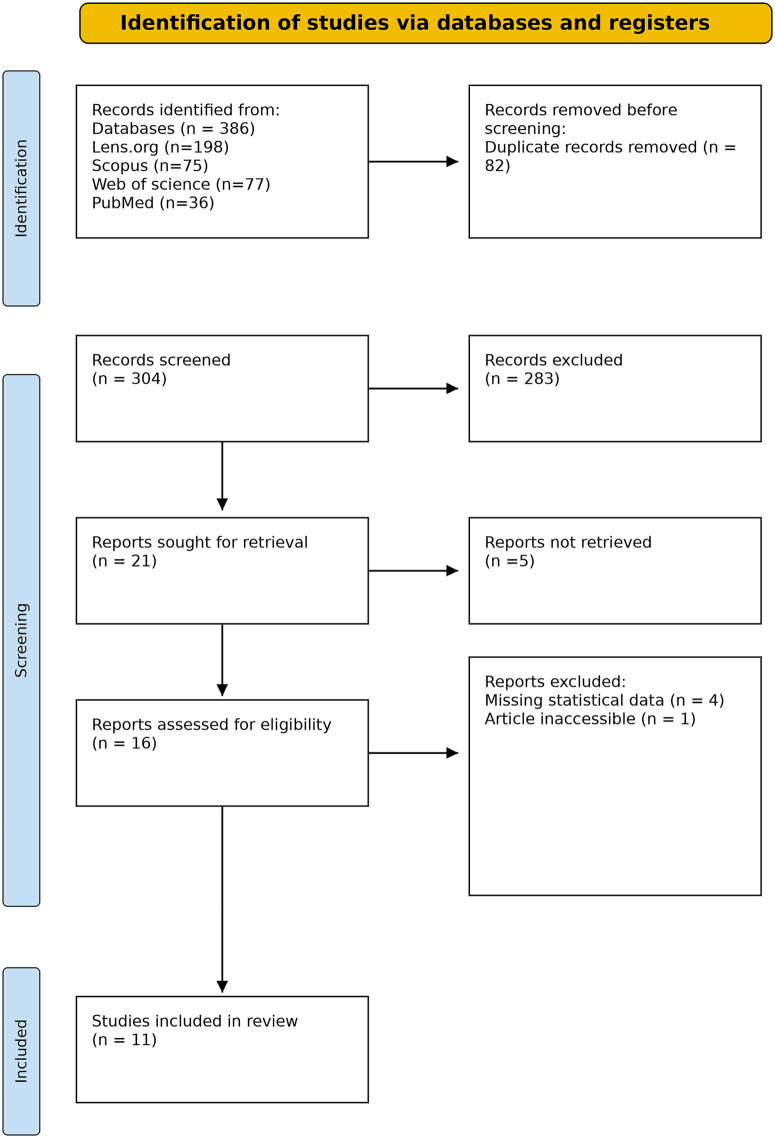
PRISMA flow diagram demonstrating the article screening and selection process.

A total of 386 articles were initially obtained from the selected databases (PubMed: 36, Scopus: 75, Web of Science: 77, Lens.org: 198). A total of 82 duplicates were removed. This resulted in 304 titles and abstracts that were screened. Twenty-one articles were sought and underwent eligibility screening. Eleven studies were included in the final systematic review (Fig 1). The final studies included a total population of 1,099 women with PE from China, Nigeria, Ghana, Vietnam, Uganda, Ethiopia, and Pakistan. The prevalence of hyperuricemia in PE ranged from 36.1 to 75% (Table 1).

**Table 1:** Characteristics of included studies.

First Author (Year)	Country	Study Design	Preeclampsia Cases (n)	Hyperuricemia Cases (n)	Prevalence (%)	95% CI	Hyperuricemia Definition (mg/dL)
Luo et al. (2024) [9]	China	Observational cohort	292	180	61.64	55.44–67.57	≥7.0
Richmond et al. (2015) [6]	Nigeria	Cohort	20	15	75.0	53.8–88.8	>6.5
Enaruna et al. (2014) [11]	Nigeria	Prospective case-control	40	20	50.0	33.7–66.3	>5.0
Adu-Bonsaffoh et al. (2024) [2]	Ghana	Cross-sectional	100	61	61.0	51.4–70.6	≥6.3
Le et al. (2019) [3]	Vietnam	Cross-sectional	205	74	36.1	29.7–43.0	>6.62
Wehlie et al. (2025) [8]	Uganda	Prospective cohort	111	43	38.7	30.1–48.2	>6.0
Ugwuanyi et al. (2021) [12]	Nigeria	Case-control	51	27	52.9	39.0–67.0	>6.0
Akram et al. (2025) [7]	Pakistan	Prospective longitudinal	34	17	50.0	32.4–67.6	≥5.5
Hassen et al. (2022) [1]	Ethiopia	Comparative cross-sectional	51	36	70.6	61.8–79.4	>5.7
Lawal et al. 2020 [13]	Nigeria	Cohort	100	39	39.0	29.4- 48.6	>5.1
Ngeri et al. 2022 [14]	Nigeria	Case-control	95	63	66.3	56.8-75.8	>6.0

### Meta-analysis results

**Fig 2:**
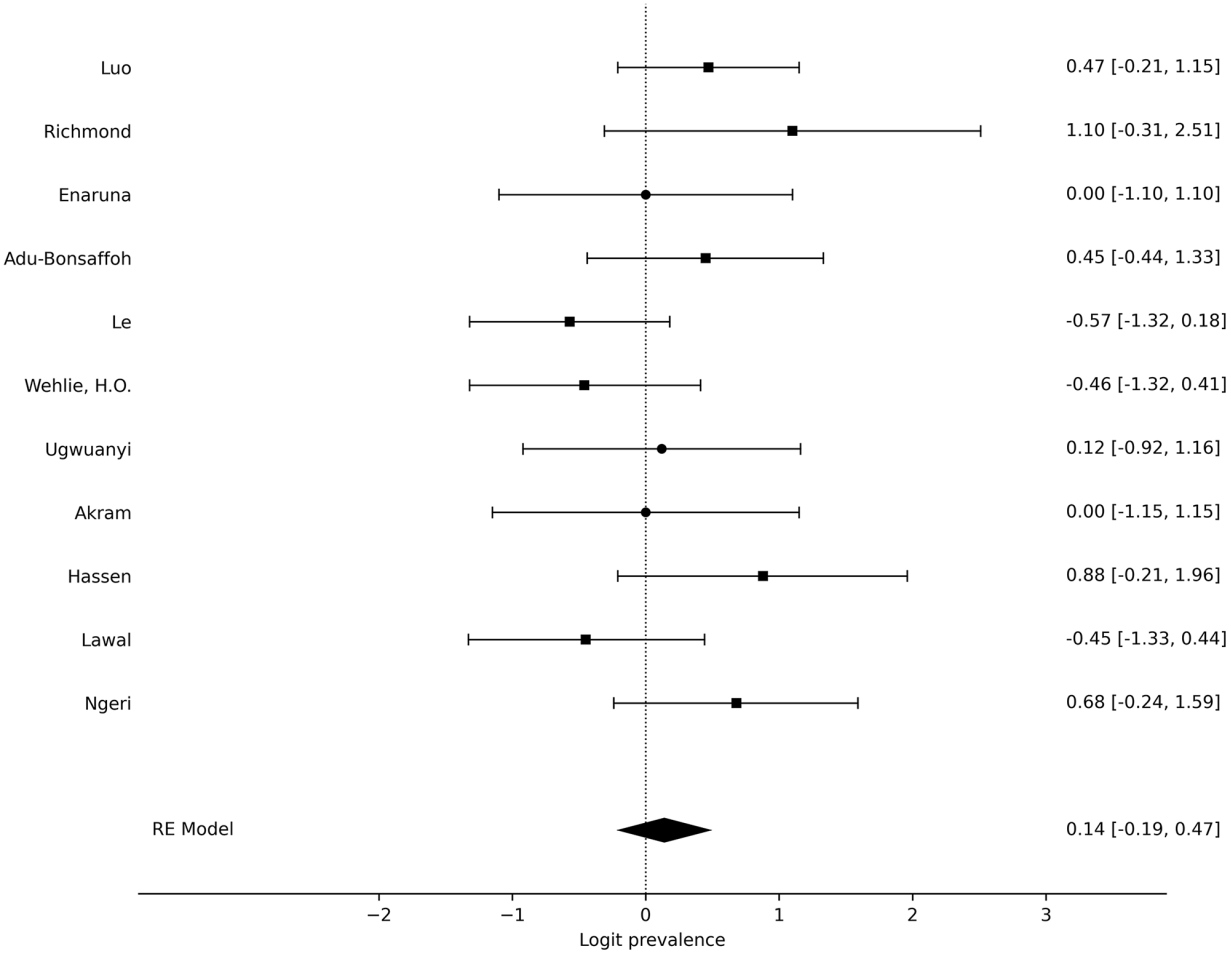
Forest plot of prevalence of hyperuricemia in pregnant women with preeclampsia. The forest plot displays prevalence estimates and 95% confidence intervals for each study, with the pooled prevalence of 53.47%. Study weights reflect sample size and precision, with low-to-moderate heterogeneity (I² = 27.47%) evident from the alignment of confidence intervals.

The random-effects meta-analysis yielded a pooled prevalence of hyperuricemia in preeclampsia of 53.47% (95% CI: 45.17% to 61.58%) (Table 2). Between-study heterogeneity was low to moderate (Tau² = 0.0854; I² = 27.47%; Q = 13.367, df = 10, p = 0.204) (Table 3). The corresponding forest plot is shown in Fig 2.

**Table 2:** Meta-analysis results.

Parameter	Estimate	Standard Error	95% Confidence Interval	p-value
Pooled Logit Prevalence	0.1390	0.1700	−0.1940 to 0.4710	0.414
Pooled Prevalence (%)	53.47	–	45.17 to 61.58	–

**Table 3:** Heterogeneity statistics.

Statistic	Value	95% CI	p-value
Tau²	0.0854	SE = 0.1413	–
Tau	0.292	–	–
I²	27.47%	–	–
H²	1.378	–	–
Q-statistic	13.367	df = 10	0.204

### Publication bias assessment

**Fig 3:**
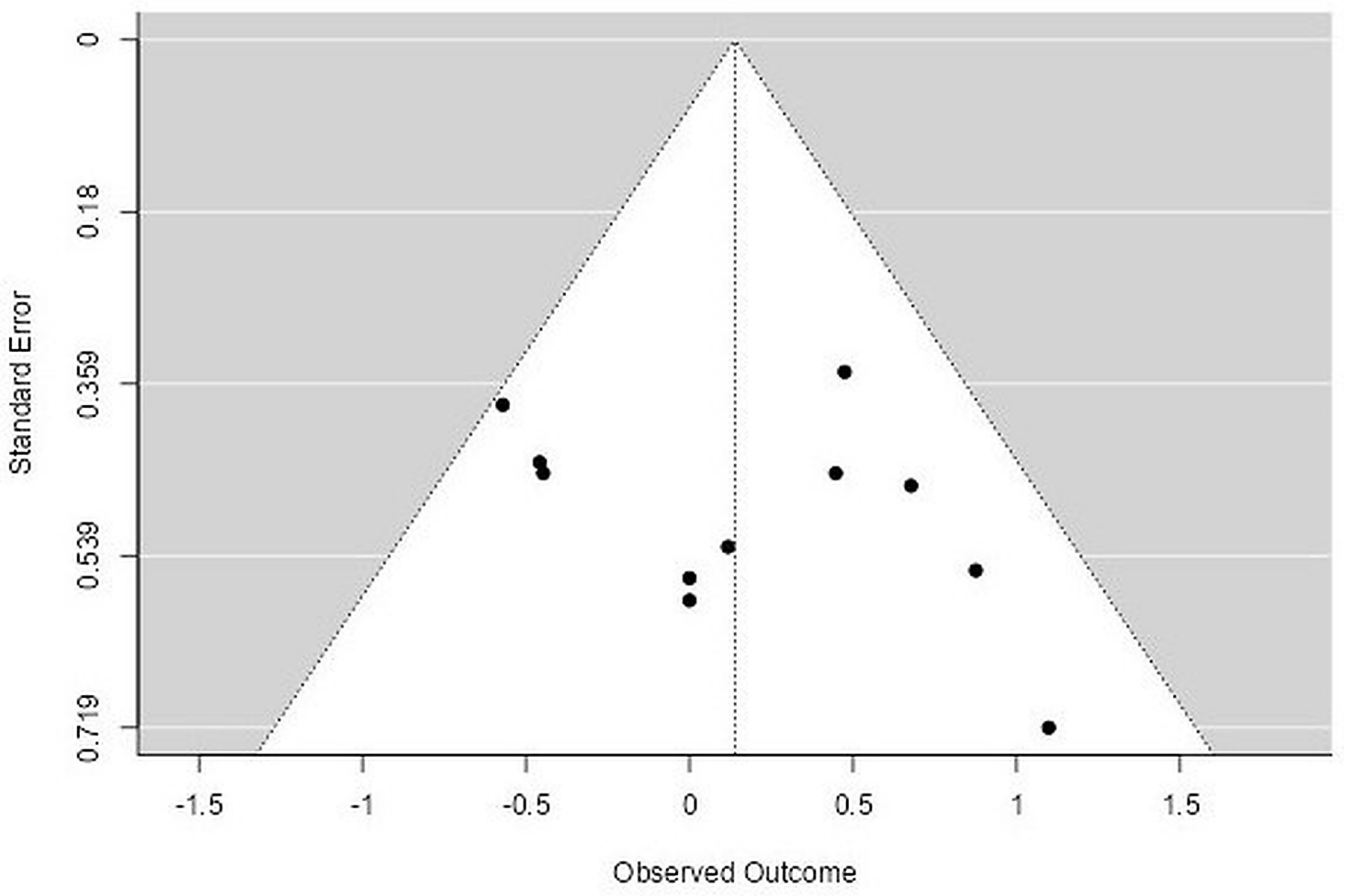
Funnel Plot of Publication Bias. The funnel plot demonstrates the study effect sizes (logit-transformed prevalence) plotted against standard errors. The symmetric distribution around the pooled estimate suggests minimal publication bias, corroborated by non-significant Egger’s (p = 0.255) and Kendall’s Tau (p = 0.160) tests.

Publication bias assessments showed no significant bias. The fail-safe N was 0 (p = not reported), indicating limited protection against bias indicating that 5 additional studies with null findings would be needed to alter the significance of our pooled prevalence estimate. However, Kendall’s Tau (0.330, p = 0.160) and Egger’s regression (1.139, p = 0.255) suggested no significant bias (Fig 3, Table 4).

**Table 4:** Publication bias assessment.

Test	Statistic	p-value	Interpretation
Fail-safe N	5.000	----	Potential vulnerability to publication bias
Kendall’s Tau	0.330	0.160	No significant small-study effects
Egger’s Regression	1.139	0.255	No significant publication bias

Exploratory small-study effect assessments were examined cautiously because the analysis pooled prevalence estimates rather than comparative effect sizes. These analyses were not considered central to interpretation of the main findings and were therefore not used to support firm conclusions regarding publication bias.

### Equivalence testing

Two one-sided tests (TOST) for equivalence with bounds of ±0.5 logit units were significant (Z = −2.643, p = 0.004), indicating the pooled effect is statistically equivalent to zero within these bounds. The null hypothesis test was non-significant (Z = 0.817, p = 0.414; Table 5).

**Table 5:** Equivalence testing results.

Test component	Z-value	p-value	Confidence interval
Lower Bound TOST	3.921	<0.001	–
Upper Bound TOST	−2.643	0.004	–
Equivalence Test	−2.643	0.004	−0.194 to 0.471
Null Hypothesis Test	0.817	0.414	−0.194 to 0.471

### Regional variation

Prevalence varied by region, with West Africa (50.0–75.0%) and East Africa (38.7–70.6%) showing higher rates of hyperuricemia than South Asia (50.0%) and Southeast Asia (36.1%; Table 6). These descriptive differences should be interpreted cautiously because the number of studies per region was small and some regions were represented by only one study

**Table 6:** Regional distribution of studies.

Region	Number of Studies	Countries (n)	Cases of Hyperuricemia (n)	Prevalence Range (%)
West Africa	6	Nigeria (5), Ghana (1)	225	50.0–75.0
East Africa	2	Uganda (1), Ethiopia (1)	79	38.7–70.6
South Asia	1	Pakistan (1)	17	50.0
East Asia	1	China (1)	180	61.64
Southeast Asia	1	Vietnam (1)	74	36.1

### Study quality assessment

Four studies were rated high quality (JBI score ≥6), with robust methodology and clear definitions. Six studies were moderate due to smaller sample sizes or methodological limitations. High-quality studies had larger sample sizes (>100) and robust methodology, while moderate-quality studies had smaller samples or less rigorous designs, potentially affecting precision (Table 7).

**Table 7:** Study quality assessment summary.

Author	Preeclampsia cases (n)	Study design score	Definition clarity	Overall quality
Luo et al. [9]	292	High	Good	High
Richmond et al. [6]	20	Moderate	Good	Moderate
Enaruna et al. [11]	40	Moderate	Fair	Moderate
Adu-Bonsaffoh et al. [2]	100	High	Good	High
Le et al. [3]	205	High	Good	High
Wehlie et al. [8]	111	High	Good	High
Ugwuanyi et al. [12]	51	Moderate	Good	Moderate
Akram et al. [7]	34	Moderate	Good	Moderate
Hassen et al. [1]	51	Moderate	Good	Moderate
Lawal et al. [13]	100	Moderate	Good	Moderate
Ngeri et al. [14]	95	Moderate	Good	Moderate

## Discussion

This systematic review and meta-analysis found that hyperuricemia was reported in approximately half of women with preeclampsia in hospital-based LMIC studies, with a pooled prevalence of 53.47% (95% CI: 45.17% to 61.58%). This finding indicates that elevated serum uric acid is a common biochemical feature among women with preeclampsia in these settings. However, because this review synthesized prevalence data only, the findings should be interpreted descriptively and should not be taken as evidence of diagnostic accuracy, prognostic value, or clinical effectiveness.

Despite differences in sample sizes, designs, and diagnostic thresholds for hyperuricemia (ranging from >5.0 to ≥7.0 mg/dL), the meta-analysis exhibited low-to-moderate heterogeneity (I² = 26.46%, p = 0.215), suggesting relative consistency across included studies. This statistical homogeneity enhances the reliability of the pooled estimate and affirms the robustness of the prevalence findings across diverse LMIC populations.

The methodological quality of included studies was generally moderate to high, with four studies scoring ≥6 on the JBI scale. Most studies applied standard clinical definitions of PE and measured serum uric acid through laboratory assays. However, all studies were conducted in inpatient hospital settings, which may skew prevalence estimates toward more severe or referred cases, possibly underrepresenting the true population-level burden.

Prevalence varied geographically, with West Africa (50.0–75.0%) and East Africa (38.7–70.6%) showing higher rates than South Asia (50.0%) and Southeast Asia (36.1%). The highest prevalence was reported in Nigeria (75.0%) [6], while the lowest was in Vietnam (36.1%) [3]. These regional differences may reflect variations in clinical criteria and diagnostic protocols, or genetic susceptibility [15]. These regional discrepancies are consistent with earlier findings that suggest differences in the burden and severity of PE across global regions, particularly in women of African ancestry and specifically in Nigeria where PE is the leading cause of maternal mortality [5,16].

The findings from individual studies further align with the pooled result. For instance, Ugwuanyi et al. reported elevated uric acid levels in preeclamptic women compared to normotensive controls, supporting the association between hyperuricemia and PE [12]. Similarly, Hassen et al. found significantly higher mean uric acid concentrations in preeclamptic women (6.17 ± 1.04 mg/dL) versus controls (3.65 ± 1.19 mg/dL, p < 0.001), with a prevalence rate of 70.6% [1]. These consistent findings across studies affirm that hyperuricemia is a common biochemical abnormality in preeclamptic pregnancies in LMICs.

The highest recorded prevalence was observed in Nigeria (75.0%) [6], and the lowest in Vietnam (36.1%) [3], highlighting possible differences in disease severity, healthcare access, or baseline maternal health [4]. While focused more globally, this data also highlights the relevance of hyperuricemia as a marker of PE severity, particularly in early-onset disease. The current LMIC-specific findings address a critical evidence gap by providing focused data from settings where the burden is high and diagnostic resources are limited.

Taken together, the pooled prevalence of 53.47% from this meta-analysis confirms that hyperuricemia is a common finding in preeclamptic pregnancies across LMICs. This finding is in line with the pathophysiological understanding that hyperuricemia in PE results from reduced renal clearance and increased oxidative stress [1,12]. Moreover, Khurshid et al. [17] and Corominas et al. [4] have reported the diagnostic utility of serum uric acid, though such aspects extend beyond the primary scope of prevalence-focused analysis.

The main contribution of this review is to synthesize hospital-based evidence from LMIC settings, where the burden of preeclampsia is high and published data are often fragmented across individual studies. Rather than establishing a new biological or clinical role for uric acid, this review quantifies how commonly hyperuricemia is reported in women with preeclampsia in these settings and highlights key methodological limitations in the available literature.

The low-to-moderate heterogeneity suggests that reported prevalence was not highly inconsistent across included studies. Nevertheless, study-level differences in design, sample size, and diagnostic thresholds may still have influenced individual prevalence estimates. In particular, the threshold used to define hyperuricemia ranged from 5.0 to 7.0 mg/dL [9], which limits direct comparability across studies and highlights the need for more standardized reporting.

Regional variation was notable, with higher rates in West and East Africa [1,6] compared to Southeast Asia [3]. Study designs also influenced precision, with prospective cohorts such as Wehlie et al. yielding more consistent estimates than retrospective studies [8]. Inconsistent gestational timing may have further contributed to variations in prevalence reporting, as levels of hyperuricemia have been shown to increase with gestational weeks [4], while others have demonstrated a negative correlation [2,11].

An important issue in interpreting the pooled prevalence is the timing of serum uric acid measurement during pregnancy. Uric acid levels vary physiologically across gestation and may rise further with increasing disease severity. Because most included studies did not report gestational age or trimester at the time of measurement, it was not possible to determine whether timing contributed to the observed prevalence or to between-study variability. This limitation is particularly relevant because all included studies were hospital-based and may overrepresent women presenting later in pregnancy, after referral, or with more severe disease.

This meta-analysis is the first to synthesize the prevalence of hyperuricemia in PE within hospitals across LMICs, addressing a critical evidence gap. It includes studies from geographically diverse LMICs, enhancing the generalizability of findings within these settings. The analysis employed random-effects modeling and the TOST approach for rigorous statistical evaluation. A comprehensive assessment of publication bias using multiple tests and a funnel plot (Fig 3) confirmed minimal bias, strengthening confidence in the results.

Generalizability is also limited by the geographic distribution of included studies. Most studies were from Africa and Asia, and no eligible studies from Latin America were identified. Accordingly, the findings should not be interpreted as representative of all LMIC settings globally.

This review has several limitations. First, all included studies were hospital-based, which may overrepresent referred, later-presenting, or more severe cases and may therefore limit generalizability beyond inpatient settings. Second, diagnostic thresholds for hyperuricemia varied across studies, reducing comparability of prevalence estimates. Third, gestational age at the time of serum uric acid measurement was inconsistently reported, preventing trimester-specific analysis and limiting interpretation because uric acid levels vary across pregnancy. Fourth, most studies did not report severity-specific or outcome-specific data, so this review could not assess associations with maternal outcomes, disease severity, diagnostic performance, or prognostic value. Finally, the geographic representation of studies was limited, with evidence drawn mainly from Africa and Asia.

### Implications for research and reporting

This review shows that hyperuricemia is commonly reported among women with preeclampsia in hospital-based LMIC studies. However, these findings do not establish whether serum uric acid has diagnostic accuracy, prognostic value, or usefulness for clinical decision-making. The main implications are methodological: future studies should adopt standardized thresholds for hyperuricemia, report gestational age at the time of testing, distinguish disease severity where possible, and evaluate clinical outcomes using prospective designs.The 52.00% prevalence is high and supports routine uric acid testing in LMICs antenatal care protocols. Hyperuricemia’s high sensitivity (65%) [17] and negative predictive value (99.5%) [4] make it a valuable tool for identifying high-risk preeclamptic patients, enabling timely interventions [8]. Policymakers should prioritize affordable uric acid assays and strengthen antenatal care infrastructure to reduce preeclampsia-related morbidity and mortality in LMICs. Training healthcare providers to interpret uric acid levels in the context of regional thresholds (e.g., > 6.0 mg/dL) could enhance risk stratification and resource allocation.

## Future research directions

This review highlights key research priorities to advance understanding of hyperuricemia in PE within LMICs. Standardizing hyperuricemia thresholds would enhance comparability and clinical relevance. Longitudinal studies are needed to explore how changes in uric acid levels relate to adverse outcomes such as preterm birth and fetal growth restriction. Future meta-analyses should focus on specific clinical outcomes to strengthen the evidence base for guidelines. Broader inclusion of underrepresented LMIC regions would improve generalizability, while therapeutic trials targeting uric acid reduction could uncover new interventions for improving maternal and perinatal outcomes.

## Conclusions

This systematic review and meta-analysis found that hyperuricemia is common among women with preeclampsia in hospital-based LMIC studies. The review provides a pooled estimate of prevalence, but it does not establish diagnostic accuracy, prognostic performance, or clinical utility. Interpretation should remain cautious because of variation in diagnostic thresholds, limited regional representation, and inconsistent reporting of gestational timing. Future research should standardize measurement and reporting and evaluate clinical and outcome-based associations prospectively.

## Supporting information

S1 File Search strategies. (DOCX)

S1 Table Joanna Briggs Institute (JBI) Quality Scores. (DOCX)

S1 Checklist PRISMA 2020 checklist. (DOCX)
