# Supplementary material for: Prevalence of hyperuricemia in preeclampsia: A systematic review and meta-analysis of studies from low - and middle - income countries
Source: PLoS One. 2026 Jun 26;21(6):e0345152. doi: 10.1371/journal.pone.0345152 (PMC13308826; doi:10.1371/journal.pone.0345152)
Supplement: S1 File — (DOCX) [file pone.0345152.s001.docx]

**Supplementary Information**

**Web of Science Search Query**

TS=((hyperuricemia OR hyperuricaemia OR "uric acid" OR "serum uric acid" OR "plasma uric acid" OR "elevated uric acid" OR "high uric acid" OR urate OR hyperuricacidemia) AND (preeclampsia OR pre-eclampsia OR "pregnancy induced hypertension" OR "pregnancy-induced hypertension" OR PIH OR "gestational hypertension" OR "hypertensive disorders of pregnancy" OR "pregnancy hypertension" OR eclampsia OR "severe preeclampsia" OR "mild preeclampsia") AND ("low income" OR "middle income" OR "developing country" OR "developing countries" OR "resource limited" OR "resource-limited" OR "low resource" OR "middle resource" OR LMIC OR LMICs OR "sub-saharan " OR "south asia" OR "southeast asia" OR africa OR asia OR "latin america" OR "south america" OR bangladesh OR india OR pakistan OR nigeria OR ethiopia OR "democratic republic of congo" OR kenya OR tanzania OR uganda OR ghana OR mozambique OR madagascar OR cameroon OR "ivory coast" OR niger OR "burkina faso" OR malawi OR zambia OR somalia OR senegal OR chad OR zimbabwe OR guinea OR rwanda OR benin OR tunisia OR "south sudan" OR burundi OR eritrea OR "sierra leone" OR togo OR libya OR liberia OR "central african republic" OR mauritania OR mongolia OR lesotho OR namibia OR botswana OR gambia OR gabon OR mauritius OR "equatorial guinea" OR swaziland OR djibouti OR fiji OR comoros OR bhutan OR "solomon islands" OR vanuatu OR "cape verde" OR samoa OR "sao tome and principe" OR tonga OR kiribati OR micronesia OR palau OR marshall OR nauru OR tuvalu OR china OR indonesia OR thailand OR "sri lanka" OR myanmar OR cambodia OR laos OR philippines OR vietnam OR nepal OR afghanistan OR tajikistan OR kyrgyzstan OR uzbekistan OR turkmenistan OR armenia OR georgia OR azerbaijan OR moldova OR ukraine OR belarus OR albania OR "bosnia and herzegovina" OR montenegro OR serbia OR macedonia OR kosovo OR turkey OR iran OR iraq OR jordan OR lebanon OR syria OR yemen OR egypt OR morocco OR algeria OR sudan OR tunisia OR libya OR brazil OR mexico OR colombia OR argentina OR peru OR venezuela OR chile OR ecuador OR guatemala OR cuba OR "dominican republic" OR haiti OR bolivia OR honduras OR paraguay OR nicaragua OR "el salvador" OR "costa rica" OR panama OR uruguay OR jamaica OR "trinidad and tobago" OR guyana OR suriname OR belize OR barbados OR "saint lucia" OR grenada OR "saint vincent" OR antigua OR dominica OR "saint kitts"))

**PubMed Search Query**

(hyperuricemia[Title/Abstract] OR hyperuricaemia[Title/Abstract] OR "uric acid"[Title/Abstract] OR "serum uric acid"[Title/Abstract] OR "plasma uric acid"[Title/Abstract] OR urate[Title/Abstract] OR hyperuricacidemia[Title/Abstract]) AND (preeclampsia[Title/Abstract] OR pre-eclampsia[Title/Abstract] OR "pregnancy induced hypertension"[Title/Abstract] OR "pregnancy-induced hypertension"[Title/Abstract] OR PIH[Title/Abstract] OR "gestational hypertension"[Title/Abstract] OR eclampsia[Title/Abstract] OR "severe preeclampsia"[Title/Abstract] OR "mild preeclampsia"[Title/Abstract]) AND ("low income"[Title/Abstract] OR "middle income"[Title/Abstract] OR "developing country"[Title/Abstract] OR "developing countries"[Title/Abstract] OR "resource limited"[Title/Abstract] OR "resource-limited"[Title/Abstract] OR LMIC[Title/Abstract] OR LMICs[Title/Abstract] OR "sub-saharan africa"[Title/Abstract] OR "south asia"[Title/Abstract] OR "southeast asia"[Title/Abstract] OR africa[Title/Abstract] OR asia[Title/Abstract] OR "latin america"[Title/Abstract] OR "south america"[Title/Abstract] OR bangladesh[Title/Abstract] OR india[Title/Abstract] OR pakistan[Title/Abstract] OR nigeria[Title/Abstract] OR ethiopia[Title/Abstract] OR kenya[Title/Abstract] OR tanzania[Title/Abstract] OR uganda[Title/Abstract] OR ghana[Title/Abstract] OR brazil[Title/Abstract] OR mexico[Title/Abstract] OR colombia[Title/Abstract] OR argentina[Title/Abstract] OR peru[Title/Abstract] OR venezuela[Title/Abstract] OR chile[Title/Abstract] OR ecuador[Title/Abstract] OR china[Title/Abstract] OR indonesia[Title/Abstract] OR thailand[Title/Abstract] OR philippines[Title/Abstract] OR vietnam[Title/Abstract] OR cambodia[Title/Abstract] OR myanmar[Title/Abstract] OR nepal[Title/Abstract] OR iran[Title/Abstract] OR turkey[Title/Abstract] OR egypt[Title/Abstract] OR morocco[Title/Abstract] OR algeria[Title/Abstract] OR sudan[Title/Abstract])

**Scopus Search Query**

( TITLE-ABS-KEY ( hyperuricemia OR hyperuricaemia OR "uric acid" OR "serum uric acid" OR "elevated uric acid" OR "increased uric acid" OR urate OR "high uric acid" ) ) AND ( TITLE-ABS-KEY ( preeclampsia OR pre-eclampsia OR "pre eclampsia" OR preeclamptic OR "pregnancy induced hypertension" OR "gestational hypertension" OR "hypertensive disorders of pregnancy" OR "pregnancy hypertension" OR PIH OR "toxemia of pregnancy" ) ) AND ( TITLE-ABS-KEY ( prevalence OR incidence OR frequency OR occurrence OR "clinical significance" OR "diagnostic value" OR "predictive value" OR prognosis OR outcomes OR "maternal outcomes" OR "fetal outcomes" OR "perinatal outcomes" OR mortality OR morbidity OR complications OR severity OR "risk factors" OR association OR correlation OR relationship OR "biomarker" OR "predictor" ) ) AND ( TITLE-ABS-KEY ( "low income" OR "middle income" OR "low-income" OR "middle-income" OR "LMIC" OR "LMICs" OR "developing countries" OR "resource limited" OR "resource-limited" OR "low resource" OR "limited resource" OR "sub-saharan africa" OR "south asia" OR "southeast asia" OR "latin america" OR "south america" OR "central america" OR "sub saharan" OR africa OR asia OR india OR pakistan OR bangladesh OR nigeria OR kenya OR uganda OR ghana OR ethiopia OR tanzania OR brazil OR mexico OR indonesia OR philippines OR vietnam OR thailand OR egypt OR morocco OR algeria OR nepal OR myanmar OR afghanistan OR cambodia OR madagascar OR mozambique OR malawi OR zambia OR zimbabwe OR cameroon OR "ivory coast" OR senegal OR mali OR "burkina faso" OR rwanda OR burundi OR sudan OR "south sudan" OR libya OR tunisia OR jordan OR yemen OR iraq OR syria OR lebanon OR palestine OR guatemala OR honduras OR nicaragua OR "El Salvador" OR Colombia OR Venezuela OR Ecuador OR Peru OR Bolivia OR Paraguay OR Uruguay OR "Sri Lanka" OR "Papua new guinea" OR Fiji OR "Solomon islands" OR Vanuatu ) ) AND PUBYEAR > 2009 AND PUBYEAR < 2026 AND ( LIMIT-TO ( SRCTYPE,"j" ) ) AND ( LIMIT-TO ( DOCTYPE,"ar" ) ) AND ( LIMIT-TO ( EXACTKEYWORD,"Human" ) OR LIMIT-TO ( EXACTKEYWORD,"Female" ) OR LIMIT-TO ( EXACTKEYWORD,"Preeclampsia" ) OR LIMIT-TO ( EXACTKEYWORD,"Article" ) OR LIMIT-TO ( EXACTKEYWORD,"Uric Acid" ) OR LIMIT-TO ( EXACTKEYWORD,"Pregnancy" ) )

**Lens.org Search Query**

(hyperuricemia OR "uric acid") AND (preeclampsia OR preeclamptic) AND (prevalence OR "clinical significance" OR "predictive value" OR "maternal outcomes" OR "perinatal outcomes" OR mortality OR morbidity OR severity OR "biomarker") AND ("low income" OR "middle income" OR "LMIC" OR "developing countries" OR "resource limited" OR africa OR asia OR india OR nigeria OR kenya OR uganda OR ethiopia OR tanzania OR brazil OR mexico OR bangladesh OR pakistan OR egypt OR nepal OR cambodia OR indonesia OR philippines)
