# Supplementary material for: Prevalence of hyperuricemia in preeclampsia: A systematic review and meta-analysis of studies from low - and middle - income countries
Source: PLoS One. 2026 Jun 26;21(6):e0345152. doi: 10.1371/journal.pone.0345152 (PMC13308826; doi:10.1371/journal.pone.0345152)
Supplement: S1 Table — (DOCX) [file pone.0345152.s002.docx]

**Supplementary Table S1: JBI Critical Appraisal Checklist Scores for Included Studies**

| **Study** | **Year** | **Q1** | **Q2** | **Q3** | **Q4** | **Q5** | **Q6** | **Q7** | **Q8** | **Q9** | **Total Score** | **Quality Rating** |
| --- | --- | --- | --- | --- | --- | --- | --- | --- | --- | --- | --- | --- |
| Luo et al. [9] | 2024 | Y | Y | Y | Y | Y | Y | Y | Y | N | 8/9 | High |
| Richmond et al. [6] | 2015 | Y | Y | N | Y | Y | N | Y | Y | N | 6/9 | Moderate |
| Enaruna et al. [11] | 2014 | Y | Y | N | Y | Y | N | Y | Y | N | 6/9 | Moderate |
| Adu-Bonsaffoh et al. [2] | 2024 | Y | Y | Y | Y | Y | Y | Y | Y | N | 8/9 | High |
| Le et al. [3] | 2019 | Y | Y | Y | Y | Y | Y | Y | Y | N | 8/9 | High |
| Wehlie et al. [8] | 2025 | Y | Y | Y | Y | Y | Y | Y | Y | N | 8/9 | High |
| Ugwuanyi et al. [12] | 2021 | Y | Y | N | Y | Y | N | Y | Y | N | 6/9 | Moderate |
| Akram et al. [7] | 2025 | Y | Y | N | Y | Y | N | Y | Y | N | 6/9 | Moderate |
| Hassen et al. [1] | 2022 | Y | Y | N | Y | Y | N | Y | Y | N | 6/9 | Moderate |
| Lawal et al | 2014 | Y | Y | N | Y | Y | N | Y | Y | N | 6/9 | Moderate |
| Ngeri et al | 2022 | Y | Y | N | Y | Y | N | Y | Y | N | 6/9 | Moderate |

Note. This table presents the quality assessment of the included studies using the Joanna Briggs Institute (JBI) Critical Appraisal Checklist for observational studies. Each study was evaluated across nine criteria (Q1–Q9), with "Y" indicating "Yes" (criterion met) and "N" indicating "No" (criterion not met). Scores ≥6 indicate high quality, while scores of 6 reflect moderate quality due to limitations such as smaller sample sizes or lack of objective exposure criteria.
